# High sodium ionic conductivity in PEO/PVP solid polymer electrolytes with InAs nanowire fillers

**DOI:** 10.1038/s41598-021-99663-5

**Published:** 2021-10-12

**Authors:** Chandni Devi, Jnaneswari Gellanki, Håkan Pettersson, Sandeep Kumar

**Affiliations:** 1 Department of Physics, Central University of Rajasthan, Ajmer, 305817 India; 2 Solid State Physics and NanoLund, Lund University, Box 118, 221 00 Lund, Sweden; 3 School of Information Technology, Halmstad University, Box 823, 301 18 Halmstad, Sweden

**Keywords:** Energy science and technology, Materials science

## Abstract

Solid-state sodium ion batteries are frequently referred to as the most promising technology for next-generation energy storage applications. However, developing a suitable solid electrolyte with high ionic conductivity, excellent electrolyte–electrode interfaces, and a wide electrochemical stability window, remains a major challenge. Although solid-polymer electrolytes have attracted great interest due to their low cost, low density and very good processability, they generally have significantly lower ionic conductivity and poor mechanical strength. Here, we report on the development of a low-cost composite solid polymer electrolyte comprised of poly(ethylene oxide), poly(vinylpyrrolidone) and sodium hexafluorophosphate, mixed with indium arsenide nanowires. We show that the addition of 1.0% by weight of indium arsenide nanowires increases the sodium ion conductivity in the polymer to 1.50 × 10^−4^ Scm^−1^ at 40 °C. In order to explain this remarkable characteristic, we propose a new transport model in which sodium ions hop between close-spaced defect sites present on the surface of the nanowires, forming an effective complex conductive percolation network. Our work represents a significant advance in the development of novel solid polymer electrolytes with embedded engineered ultrafast 1D percolation networks for near-future generations of low-cost, high-performance batteries with excellent energy storage capabilities.

## Introduction

Sodium ion batteries (SIBs) have generated immense research interest as potential substitutes for lithium ion batteries (LIBs) because they offer comparable electrochemical performance at low cost. In addition, their major component, sodium, is readily available, and they do not employ problematic metals such as cobolt^1,2^. Next-generation batteries are expected to be characterized by low cost, high safety, and high energy density. One of the main challenges in high-performance battery technology is to develop highly conductive electrolytes suitable for efficient transport of ions between the electrodes. Ceramic solid electrolytes have high ionic conductivity at room temperature^3^, but suffer from poor surface properties that result in highly resistive interfacial contacts to the electrodes restricting their practical use. In recent years, solid polymer electrolytes (SPEs) have emerged as the most promising candidates to replace conventional flammable and potentially dangerous liquid electrolytes^4–9^. The safety and stability of SIBs can be significantly improved by using an SPE because they are inert, and leakage is not an issue. Stability is further improved by mitigating harmful dendrite growth on the sodium (Na) anode^5^. Other advantages of SPEs include lower interface resistances, higher mechanical strength and flexibility, shape versatility, light weight, and low cost^5^. An SPE plays a dual role, acting as both separator and electrolyte. It is composed of a soft polar polymer matrix as a carrier for directly dissolved metal salts. The most widely studied SPEs for SIBs use a poly(ethylene oxide) (PEO) matrix filled with Na salts, e.g. NaPF_6_. For SIBs, PEO is the most widely studied polymer host because of its higher solubility for Na salts, its good structural and chemical stability, and the presence of flexible ethylene oxide segments and ether-bonded oxygen atoms^4,5^. Today’s state-of-the-art SPEs (polymer-Na salt blends) exhibit an ion conductivity of around 10^−6^ Scm^−1^, which is significantly lower than that of liquid electrolytes at room temperature and therefore restricts their use in commercial applications^2,10^. It is generally assumed that the highly crystalline structure of the polymers limits their ionic conductivity because it allows coupling between ionic motion and segmental motion and local relaxation of the polymer chains. It follows that reducing the crystallinity of polymers is the best strategy to increase their conductivity^4^. Over the years, many attempts have been made to enhance the conductivity of SPEs using a variety of approaches, including polymer blending, and adding plasticizers or ceramic nanofillers^11–13^. The main advantage of polymer blends is that their physical properties are easily controlled by changing the composition and synthesis conditions. PEO blended with polyvinylpyrrolidone (PVP) exhibits a high ionic mobility due to the amorphous matrix support by the PVP. The carbonyl group (C=O) present in the side chains of PVP forms complexes with inorganic salts. It has been reported that (PEO-PVP)_8_-NaPF_6_ solid polymer composites have the highest ionic conductivity (~ 10^−6^ Scm^−1^) and the best electrochemical properties compared to composites of PEO with other sodium salts e.g. NaClO_4_, NaF, NaI, NaBr, and NaIO_4_^10,14–18^. These composites still do not have high enough conductivity for commercial applications, but this may be improved by adding fast ionic conductor fillers. Introducing nanomaterials into SPEs is very attractive because they typically improve the electrolyte performance significantly, in addition to being simple to prepare and mix with the polymer. In the last decade, extensive studies have focused on adding nanoscale fillers such as nanoparticles (NPs) and nanowires (NWs) of Al_2_O_3_, SiO_2_, TiO_2_, ZrO_2_, and LiAlO_2_ to polymer hosts to enhance the ionic conductivity of SPEs for LIBs^19–26^. In addition to improving ion transport properties, nanofillers also improve the mechanical and electrochemical stability of the batteries. To date, there have been only very few reports on the effect of nanofillers in Na^+^-based SPEs for SIBs^9,27^. It is generally accepted that the enhanced ion conductivity observed by adding nanoscale fillers is primarily due to a reduction of the polymer crystallinity. Moreover, extensive experimental investigations focused the effects of adding ceramic nanoparticles on the conductivity have shown that a strong Lewis acid–base interaction between surface groups of the nanofillers and electrolyte ion species can increase the ion conductivity^24,25,28,29^. However, as yet, no detailed theoretical model that includes all of the mechanisms involved in ion transport through SPEs has been developed.

It was recently reported that SPEs incorporating high-aspect-ratio NWs have a higher ionic conductivity than SPEs filled with nanoparticles^20^. This improvement was attributed to the much longer continuous ion conduction pathway when NWs are present, effectively forming a percolation network. To date, most studies have involved ceramic NW fillers. The main objective of the present work is to develop a fundamental understanding of the mechanisms governing Na^+^ transport in SPEs filled with III–V semiconductor NWs. III–V semiconductor NWs are not only fundamentally interesting and important nanoscale structures for unraveling low-dimensional phenomena, but also very important for realizing next generations of advanced high-performance photonic and electronic devices^30–35^. Polymer nanocomposites with semiconductor nanofillers have already been used in diverse applications including electromagnetic interface shielding, gas and bio sensors, solar cells, and light-emitting diodes^36,37^. However, to the best of our knowledge, the electrochemical properties of SPEs filled with semiconductor NWs have not been explored previously. Recently, we reported on the synthesis of InAs NWs by a low-temperature solvothermal method^38^. Using this low-cost method, it is possible to grow high-quality NWs on a large scale and so provide a low-cost feedstock for use in SPEs. These InAs NWs, grown by a solvothermal method, are almost insulating (Resistance ~ GΩ) at room temperature^38^, which hinders the possibility of electronic conduction in the electrolyte. The addition of semiconductor NWs to SPEs is expected to bring new insights and develop our fundamental understanding of electrochemical interactions on a molecular scale, and of novel Na^+^ transport mechanisms. In this work, we report the results of a systematic in-depth investigation of the Na^+^ conduction efficiency in novel SPEs prepared by adding different amounts of InAs NWs to a PEO-PVP-NaPF_6_ composite polymer.

## Experimental

### Synthesis of InAs NWs

**Figure 1:**
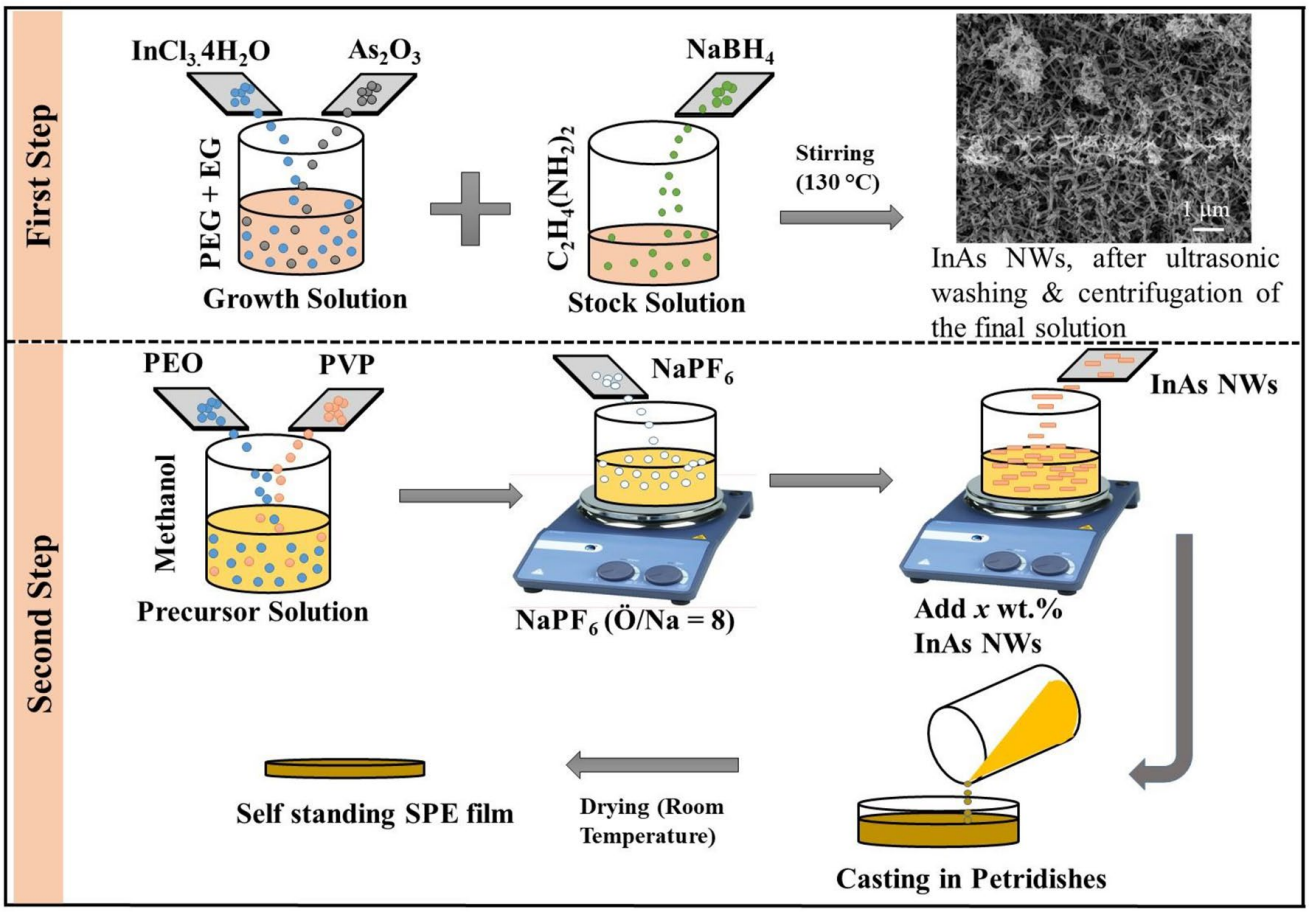
Schematic of the synthesis of InAs NWs (first step) and NW-filled composite SPEs (second step).

Single-crystalline InAs NWs of average diameter ~ 40 nm were synthesized by a low-temperature solvothermal method^38^ (Fig. 1, First Step). Indium trichloride (InCl_3_.4H_2_O) and arsenic oxide (As_2_O_3_) were used as the reactants for the InAs NW synthesis. Polyethylene glycol (PEG) and ethylene glycol (EG) were used as solvents. In this growth step of InAs NWs, InCl_3_.4H_2_O and As_2_O_3_ were used as the source for In and As, respectively. The growth solution was prepared by dissolving 200 mg As_2_O_3_ and 400 mg InCl_3_·4H_2_O in a mixture of 100 ml PEG and 50 ml EG. This solution was heated at 130 °C with continuous stirring for 30 min. A stock solution was prepared by dissolving 300 mg sodium borohydride (NaBH_4_) in 10 ml ethylenediamine. The stock solution was subsequently mixed with the growth solution. The solution was ultrasonicated and centrifuged after 1 h of reaction. The high-quality InAs NWs were finally obtained after drying at 50 °C in vacuum for 24 h. Figure S1 in the “Supporting Information” includes an FESEM image of the InAs NW network (a), XRD data (b) and an HRTEM image of a single InAs NW confirming a high-quality zincblende (ZB) crystal structure with a few stacking faults (c).

### Fabrication of SPE film

For fabrication of the SPEs (Fig. 1, Second Step), InAs NWs with 0.5–3.0 wt% were added to a methanol solution containing PEO (0.4 g), PVP (0.1 g) and NaPF_6_ salt. The molar ratios of O/Na in all the samples were held at 8:1. This mixture was mechanically stirred for 5 h and then cast into polypropylene Petri dishes. Finally, the InAs NW-filled composite SPE was dried at room temperature in vacuum overnight to completely remove all traces of the solvent.

### Characterization

The ionic conductivity was measured by AC impedance spectroscopy in the frequency range 1 Hz–1 MHz using a CHI 760 electro-chemical analyzer. An AC sinusoidal signal with amplitude 10 mV was applied to the cell with configuration Stainless steel (SS)|SPE|SS. The electrochemical stability window (ESW) was obtained by linear sweep voltammetry. For characterization of the crystallinity of the NWs and SPEs, X-ray diffraction (XRD, Bruker D8 Advance) was performed using Cu–K_α_ radiation (λ = 1.54 Å) in a Bragg angle range (2θ) from 10° to 60°.

## Results and discussion

**Figure 2:**
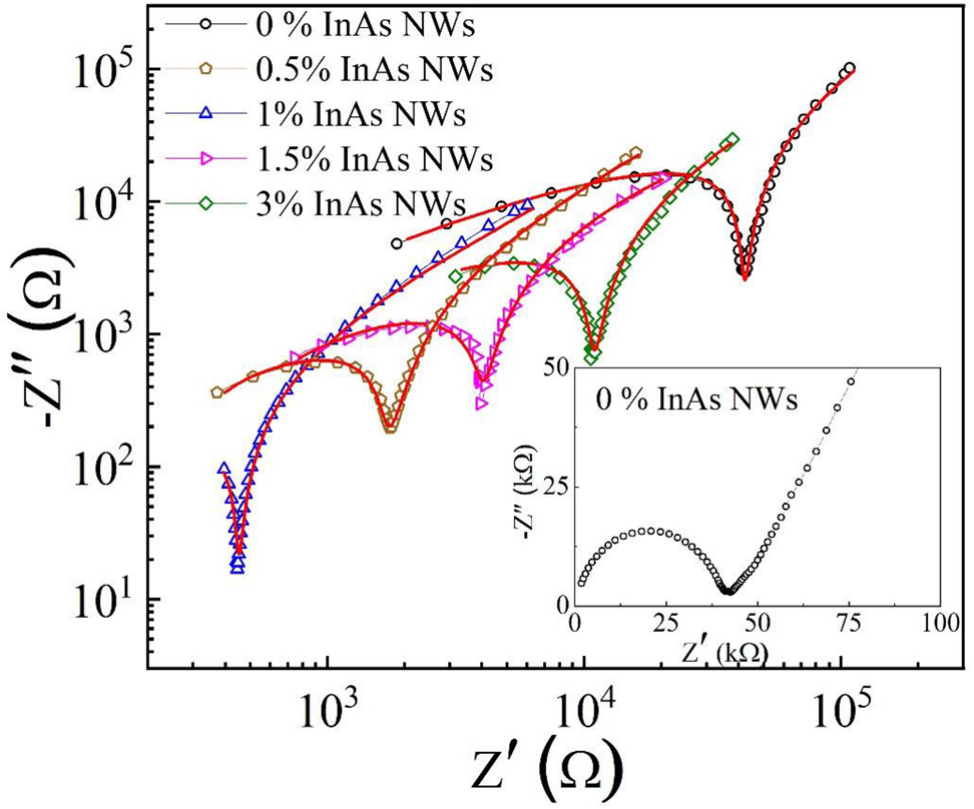
Impedance spectra of the investigated composite PEO-PVP-NaPF_6_ SPEs filled with InAs NWs plotted on a double logarithmic scale. The inset shows an impedance spectrum for the SPE without InAs NWs on a linear scale. The red lines are simulated admittance fits to the experimental data.

Ionic conductivity is the key parameter for assessing Na^+^ migration efficiency in composite SPEs. Here, this has been investigated with electrochemical impedance spectroscopy (EIS) using two stainless steel blocking electrodes. The Nyquist plots (frequency range 1 Hz–1 MHz) recorded at 40 °C of the composite SPEs with different InAs NW content (% by weight) are shown in Fig. 2.

**Figure 3:**
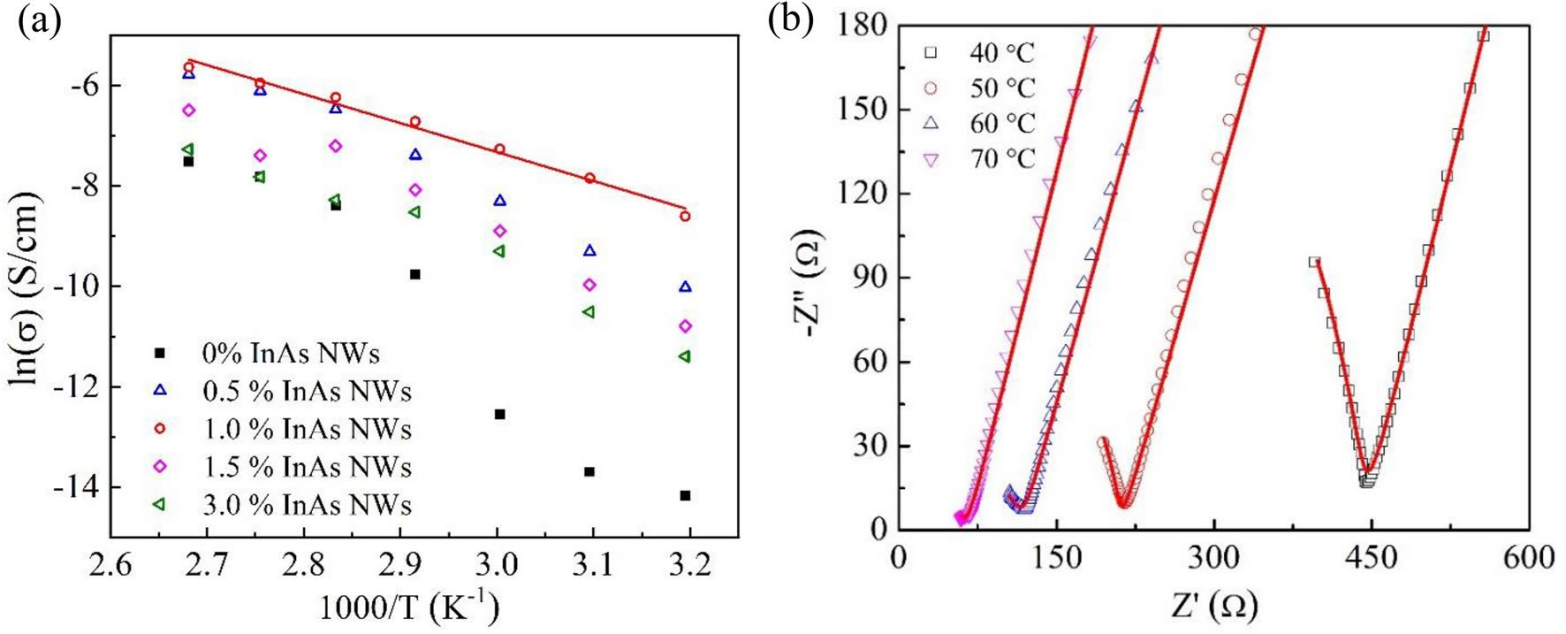
(**a**) Arrhenius plots of the extracted ionic conductivity of the SPEs. (**b**) Impedance spectra at different temperatures for the SPE with 1.0 wt% InAs NWs. The red solid lines are simulated admittance fits to the experimental spectra.

The appearence of a well-defined semicircle at high frequencies in SPEs without InAs NWs can be attributed to a combination of bulk resistance and bulk capacitance in parallel, originating from the migration of ions and polymer chains. The intercept of the semicircle with the Z′ axis yields the bulk resistance. The inset of Fig. 2 shows a typical impedance spectrum for an SPE without InAs NWs. Interestingly, Fig. 2 shows that by adding 0.5 wt% InAs NWs to the SPE the bulk resistance decreases from 40 to 2 kΩ, decreasing further to 440 Ω in an SPE with 1.0 wt% InAs NWs. It should be noted that a semicircle was not observed for the SPE with 1.0 wt% InAs NWs. This shows that there is a negligible charge transfer resistance, which in turn implies a kinetically fast electrochemical system. The sharp dip corresponds to the double layer capacitance formed at the interface between the electrodes and solid electrolyte. The temperature-dependent ionic conductivity of the different electrolytes, σ, can be extracted from the corresponding bulk resistance R_b_ using the equation:

1 $$\sigma =\frac{t}{{R}_{b}A} ,$$

where *t* is the thickness of the SPE, and A is the effective area of the blocking electrode. The temperature dependence of the ionic conductivity of the studied SPEs is shown in Fig. 3a. The composite electrolyte containing 1.0 wt% InAs NWs displayed the highest conductivity of 1.50 × 10^−4^ Scm^−1^ at 40 °C, about three orders of magnitude higher than that of the SPE without NWs and to the best of our knowledge the highest reported conductivity of any comparable SPE for Na ion batteries to date. Figure 3b shows temperature-dependent impedance spectra for the SPE doped with 1.0 wt% InAs NWs. The relationship between ionic conductivity and temperature for an SPE is typically interpreted in terms of Arrhenius-type hopping processes in conjuction with segmental polymer chain motion described by the Vogel–Tammann–Fulcher (VTF) model^28,39^. Figure 3a reveals two different conductivity regimes for the polymer without NWs. At temperatures well below the melting point, both crystalline and amorphous phases co-exist, while the amorphous phase dominates at temperatures above the melting temperature. After addition of 1.0 wt% InAs NWs, the temperature dependence of the conductivity changes to a typical Arrhenius relation over the full temperature range:

2 $$\sigma (T)={\sigma }_{0}\mathrm{exp}\left(-\frac{{E}_{a}}{kT}\right),$$

with an activation energy *E*_*a*_ of about 50 kJ mol^−1^. Moreover, the preexponential factor in Eq. (2) decreases significantly after the addition of NWs, which leads to an overall strongly enhanced conductivity. A deeper discussion of the interpretation of the large variation in activation energy can be found below.

**Figure 4:**
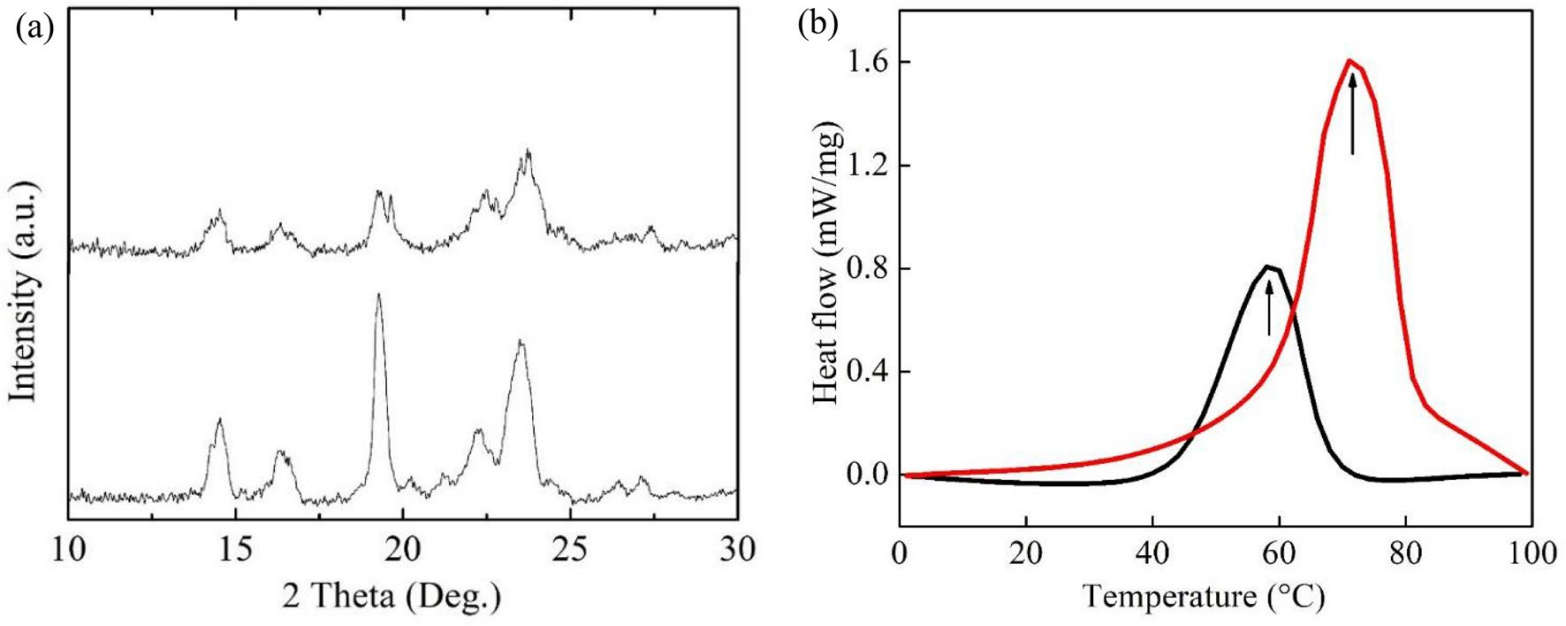
(**a**) XRD patterns for PEO-PVP -NaPF_6_ SPEs without (lower trace) and with 1.0 wt% InAs NWs (upper trace). (**b**) Corresponding DSC curves for SPE films with 1.0 wt% InAs NWs (black trace) and without NWs (red trace). The arrows indicate the respective melting temperature.

As already mentioned, one explanation for the reported enhanced conductivity of SPEs with the addition of nanofillers is a reduction in polymer crystallinity and an increase in the flexible local chains in the amorphous phase^10^. This explanation is in agreement with Fig. 3a. Figure 4a shows the room-temperature XRD patterns for PEO-PVP-NaPF_6_ SPEs without InAs NWs and filled with 1.0 wt% InAs NWs, respectively. The strong peaks at around 19° and 23.5° are attributed to the characteristic diffraction peaks from the (120) and (112) planes of semi-crystalline PEO^40^.

The decrease in amplitude of both these peaks after addition of 1 wt% InAs NWs supports the conclusion above that the added NWs indeed have a plasticizing effect on the PEO, leading to a reduction in its crystallinity. Furthermore, Fig. 4a shows that the typical diffraction peaks near 20° and 22° reported for NaPF_6_^14^ are absent in SPE films, indicating that no agglomerated salt particles exist in the composite electrolytes. The broad peak around 22°, and other additional peaks in Fig. 4a, are attributed to the formation of complexes between NaPF_6_ and PEO-PVP. The splitting of the characteristic PEO peak (19° and 23.5°) after the addition of NWs can be attributed to a conformation change due to a NW-polymer-salt complexation. The peaks near ~ 13° and 16° are associated with the formation of long-range order and may be due to the presence of ion multiplets.

To confirm that the InAs NWs reduce the crystallinity of the SPE, differential scanning calorimeter (DSC) curves were recorded (Fig. 4b) for PEO-PVP-NaPF_6_ films without InAs NWs and with 1.0 wt% InAs NWs. The observed peaks are attributed to the melting point of the corresponding SPE. As is readily observed, the melting point of the bare PEO-PVP-NaPF_6_ SPE is around 71 °C, which reduces to about 58 °C after addition of 1.0 wt% InAs NWs. The degree of crystallinity (*χ*_*c*_) of the SPEs can be assessed by the formula^41^

3 $${\chi }_{c}=\frac{\Delta {H}_{m}}{\Delta {H}_{PEO}}\times 100\%,$$

where *∆H*_*m*_ is the melting enthalpy of the SPE and *∆H*_*PEO*_ (203 J g^−1^) is the melting enthalpy of 100% crystalline PEO^42^. The value of *χ*_*c*_ decreases from 35.6 to 17.8% after the addition of InAs NWs, which shows that the added NWs do indeed decrease the crystallinity of PEO and thereby increase the PEO segmental mobility.

From the experimental results presented so far, it can be concluded that adding InAs NWs to the SPE decreases its crystallinity and so promotes the migration of Na^+^ ions through enhanced PEO segmental mobility. To explain the drastic improvement of ionic conductivity by nearly three orders of magnitude, we propose that two-phase interfacial (PEO-NaPF_6_ and InAs NW) Na^+^ transport pathways may play a much greater role in enhancing the ionic conductivity of composite SPEs than has been discussed to date.

In the standard transport model for PEO-PVP-based polymer electrolytes, Na^+^ diffuses in the amorphous phase with the assistance of mobile PEO chain segments. This can be understood in terms of an interaction between the Na^+^ and the electron-rich oxygen groups in PEO. A strong reduction in ionic conductivity was observed below 70 °C in Fig. 3a for the bare PEO-PVP-NaPF_6_ SPE. This is consistent with the observed phase transition at 71 °C in Fig. 4b where a recrystallization of PEO is expected to reduce segmental motion and the mobility of the Na^+^ ions below the melting point. Adding InAs NWs to the SPE reduces the melting point (Fig. 4b) and the activation energy (Fig. 3a), indicating that the addition of NWs makes the polymer chains more flexible, resulting in faster segmental motion and an increase in conductivity. Interestingly, adding InAs NWs not only changes the activation energy in Eq. (2), but also dramatically decreases the pre-exponential factor ${\sigma }_{0}$ by about two orders of magnitude over the whole temperature range, implying a dominant conduction mechanism different from random-walk diffusion of Na^+^ through the amorphous PEO. According to the Lewis acid–base theory for inorganic nanoparticle-filled SPEs, it is well known that the concentration and mobility of free Na^+^ play a crucial role in the ionic transport properties^43^. Figure S2 shows typical Fourier transform infrared (FTIR) vibrational absorbance spectra for PEO-PVP-NaPF_6_ SPEs with and without InAs NW fillers. The incorporation of InAs NWs alters the shape and position of the peaks, indicating the NWs' role in modifying the interaction between polymer and salt. The absorbance peak at around ~ 820 cm^−1^ directly reveals a strongly enhanced dissociation^14,22^ of NaPF_6_ into free ${\mathrm{PF}}_{6}^{-}$ and (Na^+^-${\mathrm{PF}}_{6}^{-}$) contact pairs in SPEs with added InAs NW fillers. Deconvoluted absorbance spectra in Figure S3 furthermore show that the ratio between the free anions and contact ion pairs increases after incorporation of InAs NWs. The FTIR data also demonstrate that an incorporation of InAs NWs alters the symmetric (~ 1085 cm^−1^) and antisymmetric (~ 1140 cm^−1^) C–O–C stretching modes of PEO, indicating an interaction between Na^+^ and the ether group of PEO.

It has been known for a long time that InAs surfaces and interfaces exhibit an electron accumulation layer in the near-interface region due to defect states which pin the Fermi level far above the conduction band edge^44^. It can thus be expected that the negatively-charged surface of the embedded InAs NWs in the present SPEs both promotes dissociation of contact ion-pairs, in agreement with the FTIR results discussed above, and provides a fast pathway for Na^+^ diffusion via hopping from one negatively charged site to the next, leading to an increased ion mobility. We therefore propose that the observed drastic conductivity enhancement in InAs NW-filled PEO-PVP-NaPF_6_ composite SPEs is mainly due to the formation of a hitherto unrevealed fast-conductive network induced by the unique NW surface morphology. Interestingly, the conductivity of the SPEs reaches a maximum at 1.0 wt% InAs, after which it drops with increasing InAs NW content, showing a percolation behaviour^45^. This reduction in conductivity is attributed to the formation of effective Na^+^ traps in SPEs with the largest number of percolating pathways, i.e. with largest NW filling. For example, NWs oriented parallel to the surfaces of the stainless steel electrodes could serve as effective Na^+^ traps since they restrict the ion motion normal to the electrode material, and hence decrease the conductivity.

**Figure 5:**
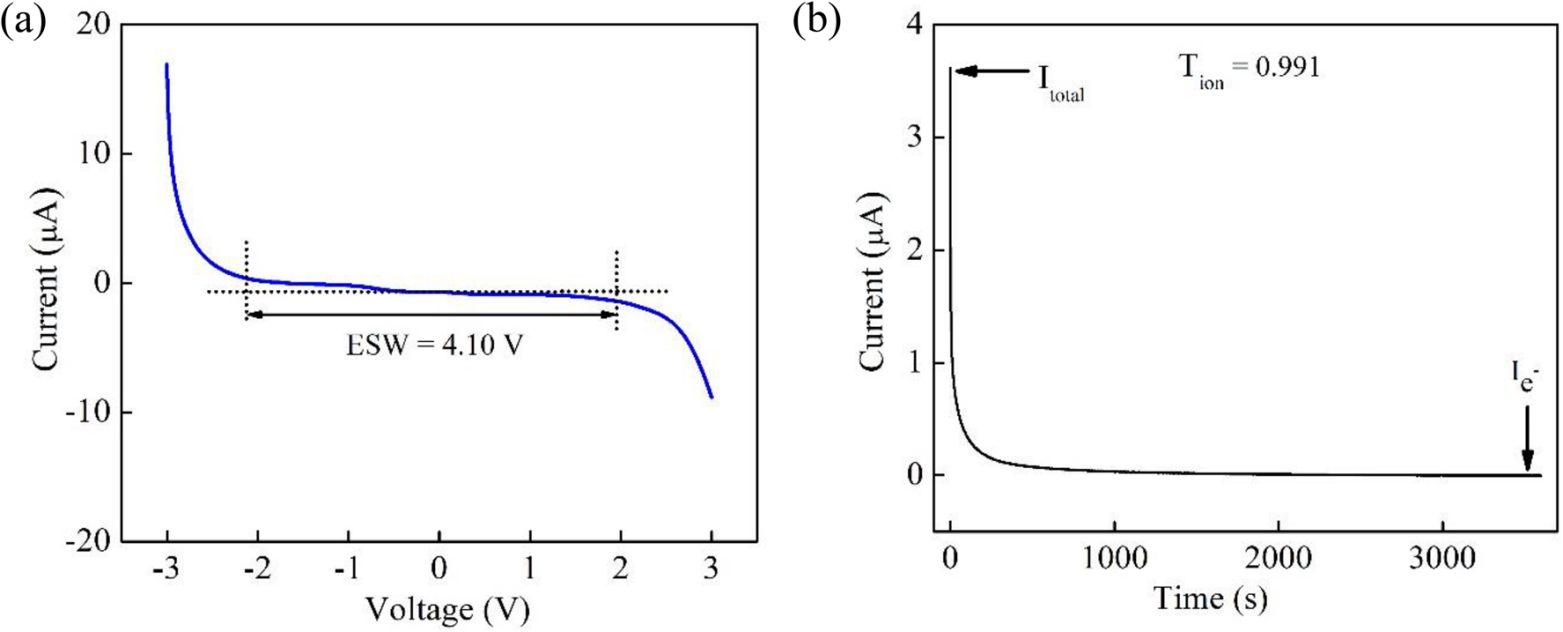
(**a**) Linear sweep voltammetry of the SS|PEO-PVP-NaPF_6_ |SS cell with 1.0 wt% InAs NWs. (**b**) Polarization current versus time for the cell in (**a**).

Besides exhibiting record-high Na^+^ conductivity, the InAs NW-filled composite SPEs also demonstrate significantly better electrochemical stability compared to SPEs without NWs. The ESW of an electrolyte is the voltage range in which the electrolyte is neither oxidized nor reduced, and it is often determined by the linear sweep voltammetry technique for the cell configuration SS|SPE|SS. The ESW for PEO-PVP-NaPF_6_ SPEs with dispersed InAs NWs was found to be 4.10 V (at 40 °C) as shown in Fig. 5a, which is sufficient for applications in high-performance energy storage devices.

In order to study the electronic and ionic contributions to the conductivity separately, we performed a transference number measurement. Here should be pointed out that this measurement cannot distinguish between anions (${\mathrm{PF}}_{6}^{-}$) and cations (Na^+^). We can however argue for a low contribution from the anions to the conductivity in these SPEs since they are immobilized by bonds to the polymer backbone as indicated by the vibrational spectra in Fig. S2 in agreement with previous reported results^14^. In contrast, the migration of Na^+^ is strongly enhanced by the segmental motion of the polymer chains which effectively disrupts the interaction between the polymer chains leaving sufficient room for Na^+^ migration. Figure 5b shows the plot of the polarization current versus time for the cell in Fig. 5a at 40 °C. The large polarization current in the early phase is due to both Na^+^ and electron transport. The current then starts to decrease rapidly with time as the stainless steel electrode blocks the ions and only allows electrons to pass. Finally, a steady-state condition is reached because of the cell polarization. To distinguish Na^+^ transport from electronic transport contributions, we have calculated the ion transference number using the relation

4 $${T}_{ion}=\frac{{I}_{T}-{I}_{{e}^{-}}}{{I}_{T}},$$

where ${I}_{{e}^{-}}$ is the electron current, and ${I}_{T}$ is the total current due to both electrons and ions. The obtained ion transference number is very close to unity (0.991) in the early phase for the studied composite SPE film. It confirms that the conductivity is mainly due to ions in agreement with the results above suggesting an effective conductive percolating network where Na^+^ hop between negatively charged defect states at the surface of the InAs NWs.

## Conclusions

In conclusion, novel composite PEO-PVP-NaPF_6_ SPEs with InAs NWs have successfully been prepared by a cheap, low-temperature, solvothermal method. Temperature-dependent Na^+^ conductivity measurements reveal a superior conductivity of 1.5 × 10^−4^ Scm^−1^ and an electrochemical stability window of 4.10 V @ 40 °C for a composite electrolyte with 1.0 wt% InAs NWs. In order to explain the excellent characteristics, we propose a new transport model where the migration of Na^+^ is strongly enhanced by hopping between nearby negatively charged defect sites present on the surface of the InAs nanowires (effective Lewis acid–base interaction) forming an effective complex conductive percolation network. Moreover, the added concentration of merely 1 wt% InAs NWs enables lightweight nanocomposites which are highly desirable in most applications. Our work represents a significant advancement in the development of novel SPEs with embedded engineered ultrafast 1D percolation networks for the next-coming generation of low-cost high-performance sodium-ion batteries.

## ﻿Supplementary Information

Supplementary Information.
