# Supplementary material for: High sodium ionic conductivity in PEO/PVP solid polymer electrolytes with InAs nanowire fillers
Source: Sci Rep. 2021 Oct 12;11:20180. doi: 10.1038/s41598-021-99663-5 (PMC8511152; doi:10.1038/s41598-021-99663-5)
Supplement: Supplementary file 1 — Supplementary Information. [file 41598_2021_99663_MOESM1_ESM.pdf]

Supporting Information for

High sodium ionic conductivity in PEO/PVP solid polymer electrolytes with InAs nanowire fillers

Chandni Devi[1], Jnaneswari Gellanki[1], Håkan Pettersson[2, 3, *] and Sandeep Kumar[1, *]

[1]*Department of Physics, Central University of Rajasthan, Ajmer-305817, India*

[2]*Solid State Physics and NanoLund, Lund University, Box 118, SE-221 00 Lund, Sweden*

[3]*School of Information Technology, Halmstad University, Box 823, SE-301 18, Halmstad, Sweden*

* corresponding authors: hakan.pettersson@hh.se, sandeep.kumar@curaj.ac.in

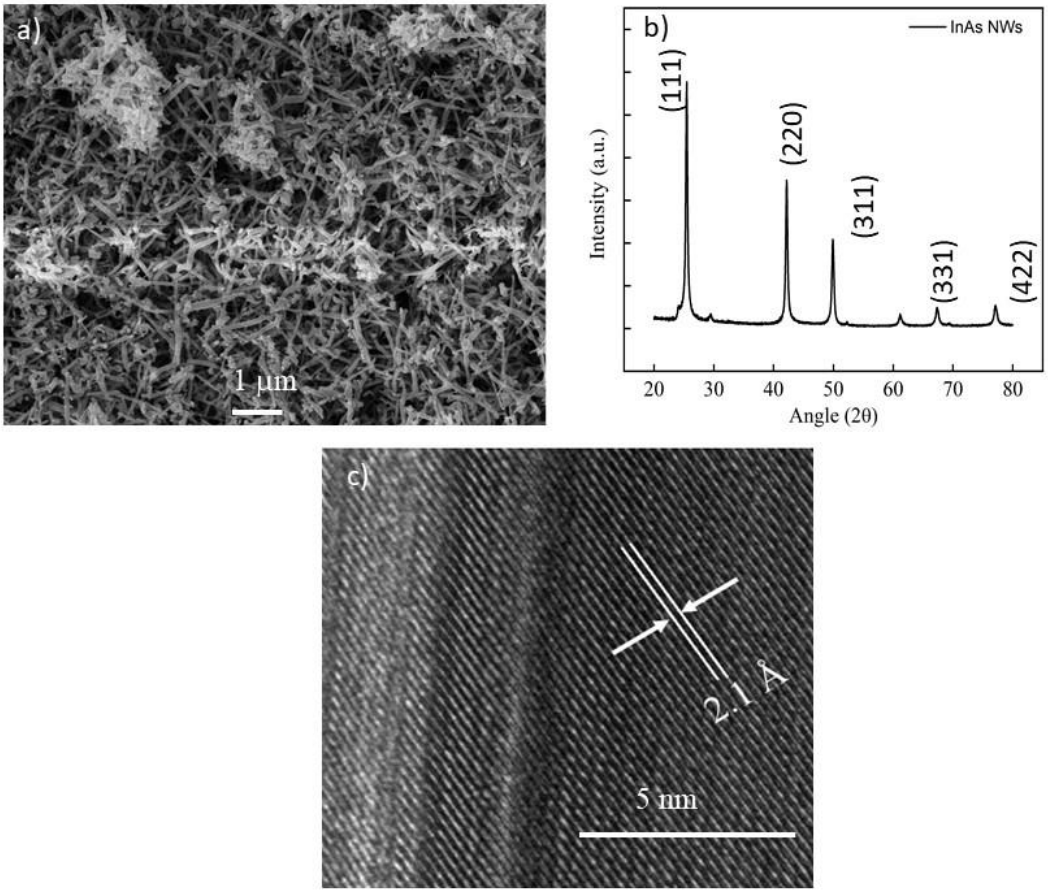

Figure S1: (a) FESEM image of a network of InAs NWs synthesized by a solvothermal method. (b) XRD spectrum of an ensemble of synthesized InAs NWs. (c). HRTEM image of a single InAs NW showing the stacking faults.

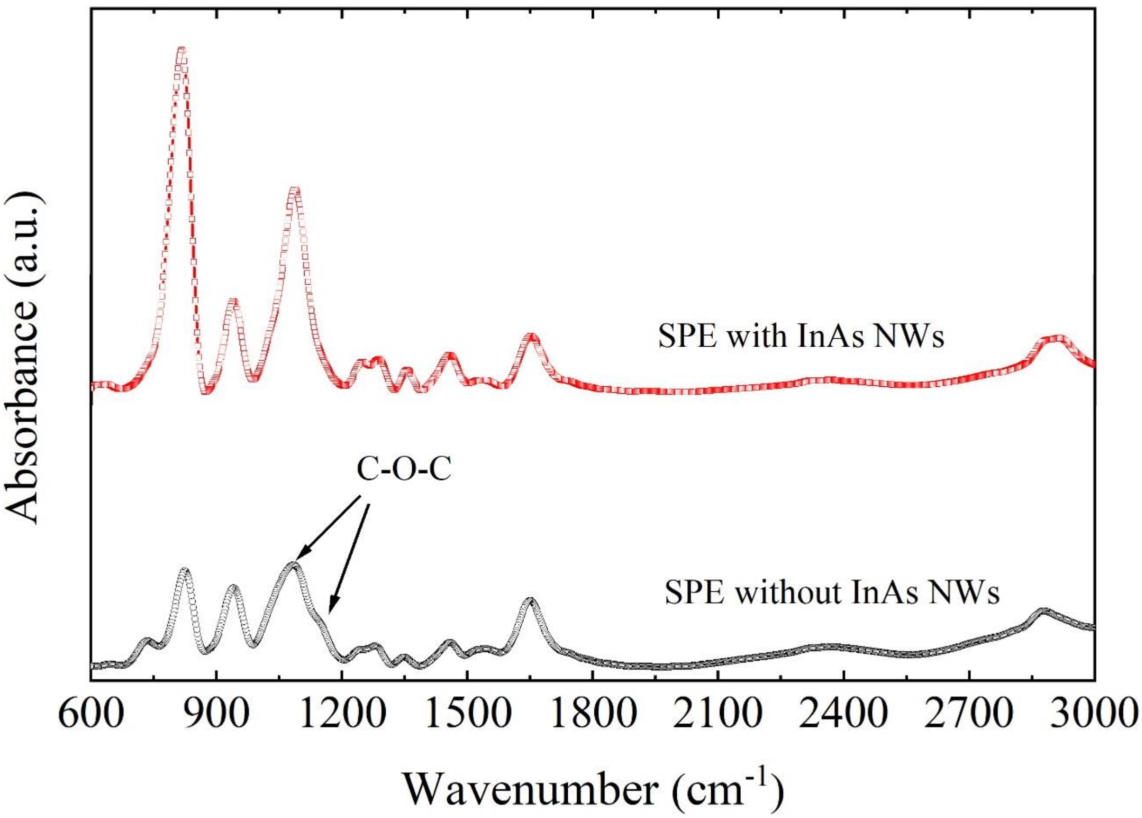

Figure S2: Absorbance spectrum of a PEO-PVP-$NaPF_6$ SPE without NWs (black curve) and with 1 wt% InAs NWs (red curve), respectively.

Figure S2 shows absorbance spectra of PEO-PVP-$NaPF_6$ SPEs with and without InAs NWs. The spectra show the main characteristic peaks of PEO and PVP in the SPE[1]. The spectra show characteristic absorbance peaks at ~1085 cm^{-1} and ~1140 cm^{-1} related to the symmetric and antisymmetric C-O-C stretching modes of PEO[1], respectively. The incorporation of InAs NWs results in an enhanced (reduced) absorbance for the symmetric (antisymmetric) mode, indicating an interaction between Na^+ and the ether group of PEO. The peak at ~820 cm^{-1} is attributed to vibration modes related to anions (PF_6^-). The fraction of free PF_6^- and contact ion pairs ($Na^+-PF_6^-$)

can be calculated from the area of the deconvoluted peaks shown in Figure S3. The peak area ratio of free PF_6^- to contact ion pairs ($Na^+-PF_6^-$) increases for the SPE with InAs NWs, which indicates that the degree of dissociation enhances.

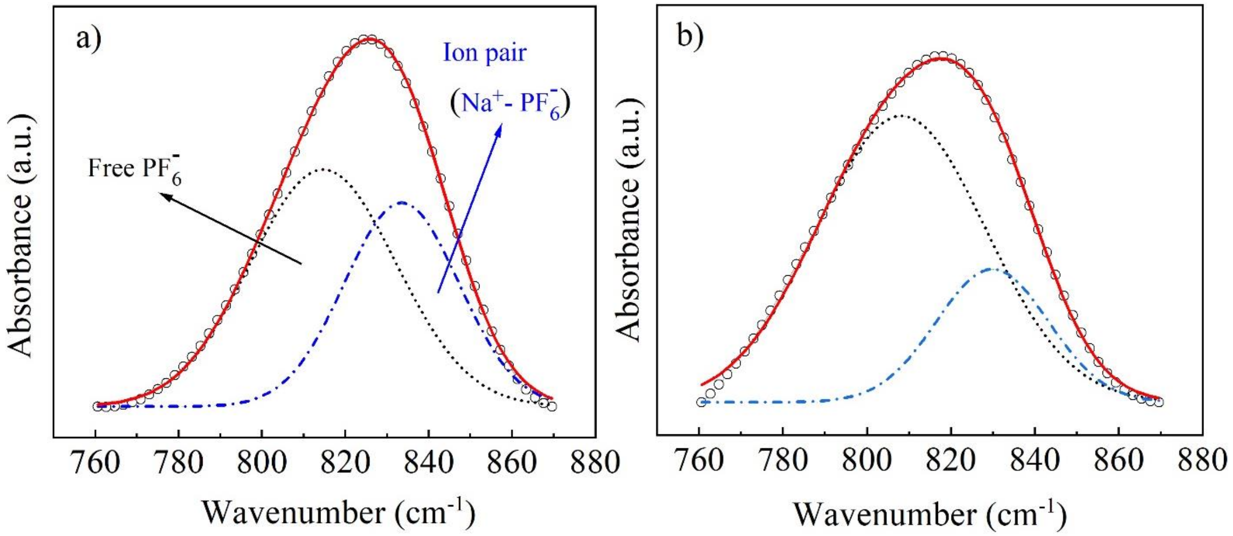

Figure S3: Gaussian-Lorentzian fittings of the free PF_6^- and (Na^+- PF_6^-) ion pair absorbance of PEO-PVP-$NaPF_6$ SPEs without a) and with b) 1 wt% InAs NWs, respectively. The red traces are fittings to the experimental data (black circles).

Reference:

1. Arya, A. & Sharma, A. L. Optimization of salt concentration and explanation of two peak percolation in blend solid polymer nanocomposite films. *J. Solid State Electrochem.* **22**, 2725–2745 (2018).
